# Electron and hydrogen self-exchange of free radicals of sterically hindered tertiary aliphatic amines investigated by photo-CIDNP

**DOI:** 10.3762/bjoc.9.46

**Published:** 2013-02-26

**Authors:** Martin Goez, Isabell Frisch, Ingo Sartorius

**Affiliations:** 1 Institut für Chemie, Martin–Luther-Universität Halle–Wittenberg, Kurt–Mothes-Str. 2, 06120 Halle/Saale, Germany

**Keywords:** amines, CIDNP, electron transfer, free radicals, hydrogen transfer, ketones, kinetics, photochemistry, self-exchange

## Abstract

The photoreactions of diazabicyclo[2,2,2]octane (DABCO) and triisopropylamine (TIPA) with the sensitizers anthraquinone (AQ) and xanthone (XA) or benzophenone (BP) were investigated by time-resolved photo-CIDNP (photochemically induced dynamic nuclear polarization) experiments. By varying the radical-pair concentration, it was ensured that these measurements respond only to self-exchange reactions of the free amine-derived radicals (radical cations **DH***^•^*^+^ or α-amino alkyl radicals **D***^•^*) with the parent amine **DH**; the acid–base equilibrium between **DH***^•^*^+^ and **D***^•^* also plays no role. Although the sensitizer does not at all participate in the observed processes, it has a pronounced influence on the CIDNP kinetics because the reaction occurs through successive radical pairs. With AQ, the polarizations stem from the initially formed radical-ion pairs, and escaping **DH***^•^*^+^ then undergoes electron self-exchange with **DH**. In the reaction sensitized with XA (or BP), the polarizations arise in a secondary pair of neutral radicals that is rapidly produced by in-cage proton transfer, and the CIDNP kinetics are due to hydrogen self-exchange between escaping **D***^•^* and **DH**. For TIPA, the activation parameters of both self-exchange reactions were determined. Outer-sphere reorganization energies obtained with the Marcus theory gave very good agreement between experimental and calculated values of ∆*G*^‡^_298_.

## Introduction

Sensitized hydrogen abstractions from tertiary aliphatic amines present a mechanistic spectrum with a varying involvement of polar intermediates. Often, they are true two-step processes with an initial full charge transfer to give a radical ion pair, which then undergoes a proton transfer [1–7]; partial charge transfer (i.e., formation of an exciplex) as the first step has also been observed [8–9].

One of the most versatile methods to elucidate complex reaction mechanisms that occur via paramagnetic intermediates is provided by measurements of chemically induced dynamic nuclear polarization (CIDNP) [10–15]. CIDNP arises from a spin-sorting process in radical pairs, which leads to opposite polarizations in the products of the two radicals of a pair with each other (geminate products) and the products of subsequent free radicals (escape products), and thus yields information about the entry and exit channels of the radical pairs. The spin sorting is driven by magnetic (i.e., Zeeman and hyperfine) interactions, completed during the pair life (i.e., on a subnanosecond timescale), and detected by NMR in the diamagnetic reaction products, where it persists for a time on the order of the nuclear *T*_1_ (i.e., a few seconds for protons). In consequence, the CIDNP effect encodes the individual hyperfine coupling constants of the nuclei in a paramagnetic intermediate as individual polarization intensities of those nuclei in a product, the so-called polarization pattern [16]; not only is this obviously useful for the identification and characterization of the intermediate but it also establishes the chemical pathways between that intermediate and the resulting products. The disparity of timescales between CIDNP generation, subsequent chemical processes, and detection opens up the possibility of time-resolved photo-CIDNP experiments [17–20]: The flash of a pulsed laser triggers a photoreaction; after a short delay, the polarizations of the products are probed with an NMR pulse; variation of the delay yields the kinetics. The method is very well suited to study bimolecular reactions of the free radicals, because typical NMR pulses are of microsecond duration and, thus, fall within the relevant kinetic range.

**Scheme 1:**
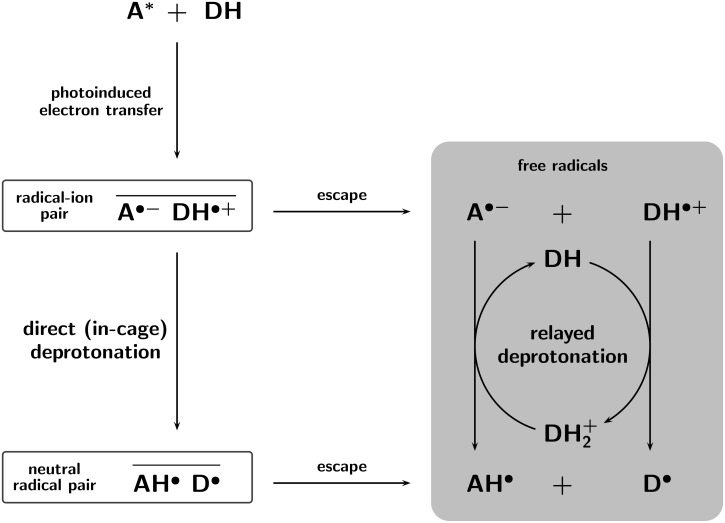
Mechanism explaining the CIDNP effects in sensitized hydrogen abstractions from tertiary aliphatic amines **DH**.

In a series of previous CIDNP studies on triethylamine with different aromatic carbonyl compounds as sensitizers [5–7], we have used the dependence of the polarization pattern on the sensitizer and the solvent to show that these reactions are always two-step hydrogen abstractions according to Scheme 1. The source of the polarizations can be either the initially formed radical-ion pair 
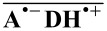
 where **A**^•−^ and **DH**^•+^ are the radical anion of the sensitizer **A** and the radical cation of the amine **DH**, or a secondary pair of neutral radicals 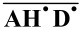, where **AH**^•^ and **D**^•^ denote the sensitizer ketyl radical and the α-amino alkyl radical. The reason why some sensitizers yield polarizations that stem from the radical ion pair, even though the precursor to the products must be the neutral radical **D**^•^, is the existence of two deprotonation pathways of **DH**^•+^: The proton can be taken up by the sensitizer radical anion in a direct reaction within the cage, or in a relayed reaction outside the cage, with surplus amine and its protonated form 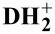 functioning as mediators. The competition between in-cage deprotonation and escape from the radical-ion pair determines the source of the polarizations. When escape predominates, all polarizations originate from the radical-ion pairs, when the in-cage deprotonation prevails, from the pairs of neutral radicals. Because the rate of in-cage deprotonation depends on the free energy of that reaction ∆*G*_dep_, a threshold behaviour is observed: For triethylamine, a complete changeover of the polarization source occurs within a narrow (<20 kJ/mol) window of ∆*G*_dep_ far in the exergonic range (at around *−*100 kJ/mol).

In this work, we employ time-resolved CIDNP experiments to study two amines with hindered deprotonation of 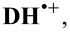 1,4-diazabicyclo[2.2.2]octane (DABCO) and triisopropylamine (TIPA). The hindrance is due to a stereoelectronic effect with DABCO [21], and due to overcrowding with TIPA [22]. As sensitizers, we have chosen 9,10-anthraquinone (AQ) on one hand and xanthone (XA) or benzophenone (BP) on the other; with triethylamine, these are typical representatives that yield CIDNP from the radical-ion pairs and from the pairs of neutral radicals, respectively. Owing to competing side-reactions, kinetic studies were not feasible with triethylamine, but for the two amines of this work, they are. As we will show, depending on the sensitizer, different spin-polarized free radicals (**D**^•+^ or **D**^•^) escape from the pairs, and then undergo self-exchange with **DH** with different rates, which can be measured by the CIDNP decay kinetics. To the best of our knowledge, this is the first comparison of the electron and hydrogen self-exchange of the same substrates.

## Results and Discussion

The relevant thermodynamic parameters of the sensitized hydrogen abstractions have been compiled in Table 1. As is evident from these values, the primary electron transfer is always so exergonic as to make it diffusion-controlled. In-cage deprotonation is also strongly exergonic, with the higher steric hindrance of TIPA being compensated by a more negative (by about 50 kJ/mol) ∆*G*_dep_; for the two classes of sensitizers, the differences of ∆*G*_dep_ even amount to as much as 60…70 kJ/mol.

**Table 1:** Triplet energies *E*_T_ of the sensitizers, energies of the radical-ion pairs *E*_RIP_, and energies of the pairs of neutral radicals *E*_NRP_ for the sensitizer/amine combinations used in this work. All energies are in kJ/mol and relative to the ground-state energies of the sensitizer plus the amine.

sensitizer/amine	*E*_T_	*E*_RIP_^a^	*E*_NRP_^b^

AQ/DABCO	247^c^	146	79
AQ/TIPA	247^c^	156	36
XA/DABCO	310^d^	225	97
XA/TIPA	310^d^	235	53
BP/DABCO	287^e^	232	91

^a^Calculated from the reduction potentials Φ_red_ vs SCE in acetonitrile or DMF; Φ_red_ (AQ) = *−*0*.*94 V [23], Φ_red_ (XA) = *−*1*.*76 V [24], Φ_red_ (BP) = *−*1*.*83 V [24], Φ_red_ (DABCO^•+^ ) = *−*0*.*57 V [25], Φ_red_ (TIPA^•+^ ) = *−*0*.*68 V [8]. ^b^From the differences of the heats of formation of the neutral radicals and their parent compounds, as calculated by Gaussian 09 [26] with the AM1 Hamiltonian. ^c^See [27]. ^d^See [28]. ^e^See [29].

In experiments with continuous illumination, these systems exhibit practically no CIDNP; in time-resolved experiments, however, they yield strong CIDNP signals, which decay to zero, or to a small fraction of their initial value, on a microsecond timescale. This situation is typical for an exchange cancellation [30]: Geminate recombination of radical pairs 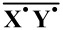 regenerates the starting materials **X** and **Y** with their respective polarizations, and escape affords free radicals **X**^•^ and **Y**^•^ bearing polarizations of exactly the same magnitudes but opposite signs to the geminate ones, owing to the spin-sorting nature of the CIDNP effect. By an exchange reaction of the free radicals with surplus reactants, e.g., **X**^•^ + **X** 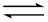 **X** + **X**^•^, the escape polarizations are then also transferred to the diamagnetic species **X** observable by NMR, and compensate the geminate polarizations already present in them. Hence, no CIDNP persists in **X** on long time scales, but the polarizations of **X** can be detected as transient phenomena because of the disparity of the timescales involved (geminate reactions are completed within nanoseconds, whereas the exchange reactions typically occur on a microsecond timescale for millimolar substrate concentrations). Residual signals remain only when the perfect neutralization of geminate and escape polarizations is disturbed by nuclear-spin relaxation in the free radicals or by secondary reactions of them.

**Figure 1:**
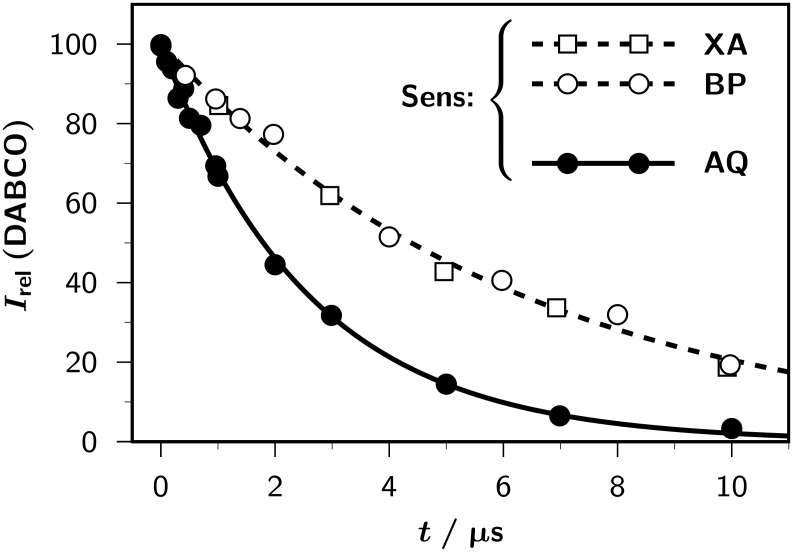
Time-resolved CIDNP in sensitized (sensitizers xanthone (XA), benzophenone (BP), or anthraquinone (AQ)) photoreactions of 1,4-diazabicyclo[2.2.2]octane (DABCO). Shown are the relative CIDNP intensities (integrals) *I*_rel_ (DABCO) of the 12 equivalent amine protons (s, 2.64 ppm) as functions of the delay *t* between the laser flash (80…90 mJ) and the sampling NMR pulse (duration, 1*.*0 µs). Temperature, 298 K; amine concentration *c*_0_, 2*.*77 *×* 10*^−^*^3^ M; sensitizer concentrations, 1 *×* 10*^−^*^3^ M (AQ, XA) and 6 *×* 10*^−^*^3^ M (BP). The fit functions are first-order rate laws, 100exp [*−k*_ex_*c*_0_*t*]; they also include data points at longer times, which are not shown in the graph. Best-fit exchange-rate constants *k*_ex_, 5*.*7 *×* 10^7^ M*^−^*^1^s*^−^*^1^ (XA, BP) and 1*.*39 *×* 10^8^ M*^−^*^1^s*^−^*^1^ (AQ). For further explanation, see text.

Figure 1 shows such decay curves for the photoreactions of DABCO with different sensitizers. We stress that in all experiments of this work, the NMR signals of unreacted starting materials were eliminated by presaturation [31], thus the displayed signal intensities correspond to the pure polarizations. Furthermore, NMR spectra taken after a full series of time-resolved CIDNP measurements gave no indication of product formation; the remarkable photostability of the BP/DABCO system has already been reported in the literature [32]. The fast initial rise of the polarizations caused by radical-pair formation was suppressed by the use of a relatively long observation pulse, which acts as a low-pass filter and leaves unchanged the much slower subsequent CIDNP decay [33].

As follows from the described mechanism, the decay rate of the polarizations of DABCO should be completely independent of the sensitizer, because the latter is not at all involved in the exchange. For the two sensitizers BP and XA this is indeed the case, as Figure 1 shows, but in the AQ-sensitized reaction the decay is considerably faster, by a factor of more than 2.

**Figure 2:**
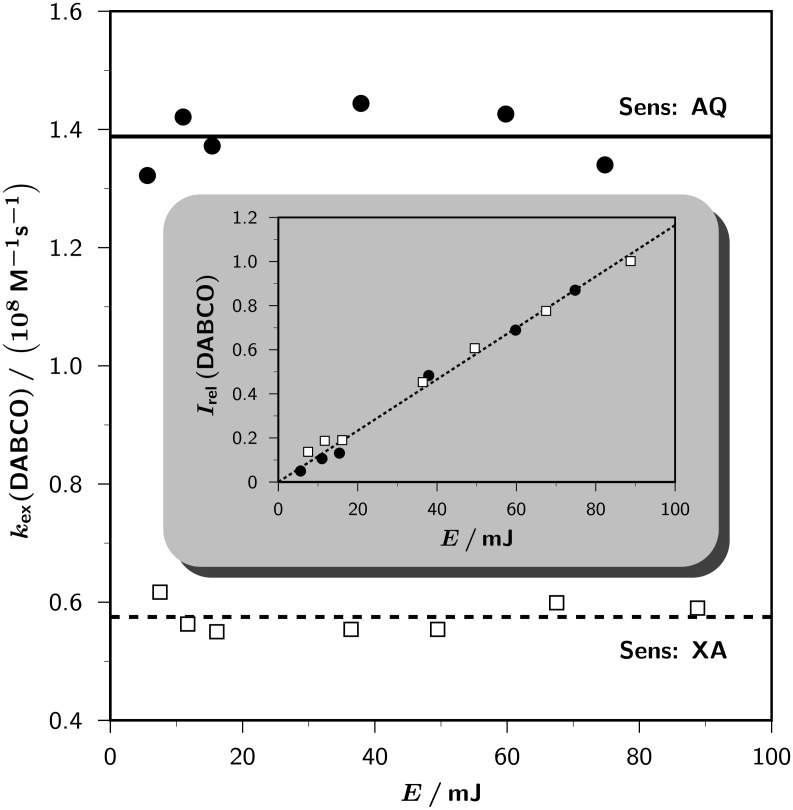
Influence of the laser intensity *E* on the observed exchange-rate constant *k*_ex_ (DABCO) (main plot) and on the relative CIDNP intensity *I*_rel_ (DABCO) of the amine protons, i.e., on the radical concentration, (inset) for the two sensitizers anthraquinone (AQ) and xanthone (XA). All other experimental parameters as in Figure 1. For further explanation, see text.

Nuclear-spin relaxation in the free radicals would increase the apparent exchange rates [34], but is ruled out by the absence of residual polarizations with AQ. This is consistent with our earlier observation of relaxation times well in excess of 100 µs for the aliphatic protons in the radical cations of methoxybenzenes [35]. The only additional pathway besides self-exchange that is capable of transferring the escape polarizations to the regenerated starting materials would be a recombination of the sensitizer-derived and amine-derived free radicals. This process is a bimolecular reaction; as a control experiment we, therefore, varied the concentrations of free radicals by varying the laser energy. The results are displayed in Figure 2. As is clearly discernible, the measured exchange rates remained constant over a concentration range spanning a factor of about 20. Because the ground states of our sensitizers can neither function as electron donors nor as hydrogen donors, exchange reactions involving different amine-derived radicals, meaning the intermediacy of different types of radical pairs, are the only remaining explanation for the different exchange rate constants for AQ as opposed to those for XA and BP. Our earlier results for triethylamine [5–7], in which time-resolved experiments are precluded by a high chemical turnover but the intermediates are identifiable through their polarization patterns, would suggest that in the present system AQ again yields CIDNP from the radical-ion pair, and the other two sensitizers produce CIDNP from the pair of neutral radicals, which is consistent with the much higher (compare, Table 1) driving force of in-cage deprotonation in the second case.

The polarization phase Γ*_i_* of nucleus *i* in a product (Γ*_i_* = +1, absorption; Γ*_i_* = *−*1, emission) is connected to details of the reaction mechanism and to the magnetic properties of the intermediates through Kaptein’s rule for a CIDNP net effect [36],

[1]
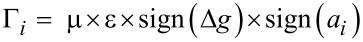


where µ and ε symbolize the multiplicities of the radical-pair precursors and the radical pairs affording the product in question (µ = +1, triplet; µ = *−*1, singlet; ε = +1, singlet; ε = *−*1, triplet), ∆*g* is the *g*-value difference of the two radicals, with the one containing nucleus *i* taken first, and *a_i_* is the hyperfine coupling constant of that nucleus in the radical. Together with the fact that the absolute CIDNP intensity of nucleus *i* is approximately proportional to *a_i_* [12], this sign rule forms the basis for identifying a paramagnetic intermediate through the resulting polarization pattern.

All three sensitizers are typical triplet sensitizers (µ = +1). From Table 1 it is evident that the energies of the resulting radical pairs lie well below those of the sensitizer triplets. The saturated amine DABCO possesses an even higher triplet energy (about 360 kJ/mol, as estimated from the phosphorescence in frozen matrices [37]) than the sensitizers, so the radical pairs can only react back to the starting materials via the singlet exit channel (ε = +1).

The DABCO signal appearing in absorption (Γ = +1) thus means that the *g*-value difference and the hyperfine coupling constant must have the same sign. For a radical ion pair, this certainly holds because the reported very high *g* value of the DABCO radical cation (2.0048 [38]) clearly exceeds the *g* values of all the sensitizer radical anions (between 2.0036 for XA [39] and 2.00443 for AQ [40]) and the proton hyperfine coupling constant in the DABCO radical cation must be positive. For a pair of neutral radicals, the situation is equally predictable although the magnetic parameters of the α-amino alkyl radical of DABCO are not known precisely: A negative hyperfine coupling constant of about *−*14 G is to be expected for the single proton attached to its radical center, a noticeably larger positive one of about +19 G for the two protons at the adjacent carbon, and a smaller positive one of about +4 G for the four *γ* protons on the other two bridges [21]; in the regenerated DABCO, all protons are magnetically equivalent, so the observed polarization phase is governed by the balance between these hyperfine coupling constants, where the positive ones clearly dominate. To account for the absorptive polarization, the *g* value of the α-amino alkyl radical must thus be larger than that of the sensitizer ketyl radical, which is corroborated by the polarizations of the regenerated sensitizer. Whereas these are undetectably small in the case of AQ and BP, XA exhibits weak polarizations of H^1^*^,^*^8^ and H^3^*^,^*^6^ (8.25 ppm, d of d; and 7.82 ppm, d of t; for the assignment, compare the literature [41]), both in absorption. H^1^*^,^*^8^ and H^3^*^,^*^6^ possess the largest hyperfine coupling constants both in the xanthone radical anion (*−*3*.*9 G for both protons [38]) and in the xanthone ketyl radical (*−*4*.*1 G for H^1^*^,^*^8^, *−*3*.*8 G for H^3^*^,^*^6^ [42]). Because of their negative signs, the *g* value of the xanthone-derived radical is thus indeed lower than that of the DABCO-derived radical.

While the polarization phases can thus be reconciled with both types of radical pairs, the different decay rates are clear evidence for different free radicals with XA as opposed to AQ. It is natural to assign hydrogen transfer to the slower of the two exchanges and electron transfer to the faster one. This is corroborated by the absolute strengths of the CIDNP effects with these two sensitizers. There is an approximate proportionality between the magnitude of CIDNP and the inverse square root of ∆*g* [11]. For the pair of radical ions, ∆*g* is about three times larger with XA than with AQ (see above). This would predict correspondingly smaller polarizations, yet quite the opposite is observed: CIDNP is more than an order of magnitude larger in the case of XA (in fact so unusually strong that for aligning the optical path of the excitation laser we found XA/DABCO to be the best system, other points in its favour being that it is extremely photostable and that its NMR signal is a singlet). This remarkable signal strength must reflect an extremely small ∆*g*, which is consistent with a pair of neutral radicals because the unknown *g* value of the α-amino alkyl radical is expected to lie only very slightly above the *g* value of the sensitizer ketyl radical (2.00345 [5]).

**Scheme 2:**
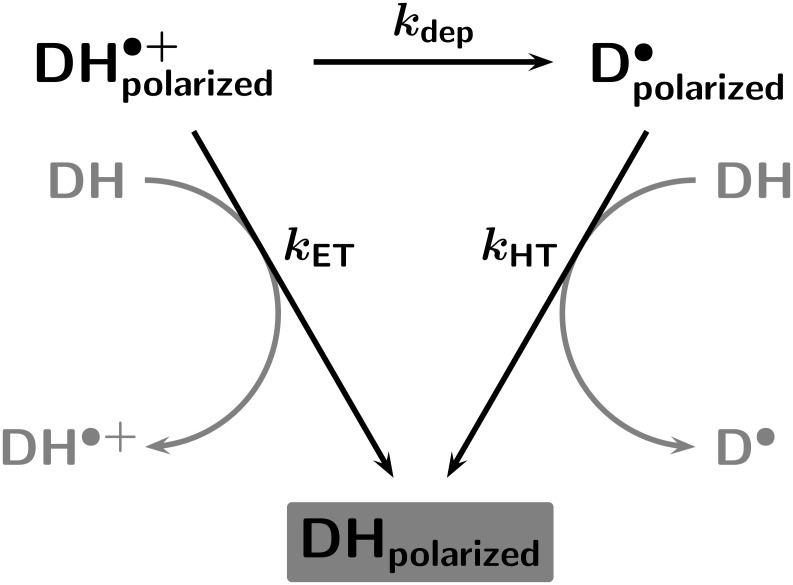
Pathways from the free radicals to the product.

To analyze the kinetics, we use Scheme 2. Polarized free radicals can escape either from the radical-ion pairs or from the pairs of neutral radicals. Each type of radical can undergo self-exchange with ground-state molecules **DH** (by electron transfer, with rate constant *k*_ET_; by hydrogen transfer, with rate constant *k*_HT_), which does not affect the chemical composition of the sample but transfers the polarizations from the radicals to **DH**, where they can be detected by NMR. We stress that there is no such thing as a polarized molecule, but that polarization is a property of the ensemble. However, because the polarizations are very small deviations of the populations of the nuclear spin states from the Boltzmann distribution (they are only noticeable compared to the tiny population differences caused by the field of the NMR magnet), the kinetics can be accurately described by a ”polarized radical” 
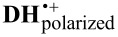
 or 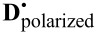 undergoing an exchange with an ”unpolarized substrate molecule” **DH** to give an ”unpolarized radical” and a ”polarized substrate molecule” **DH**_polarized_, the concentration of which is monitored. Additionally, the relayed deprotonation (rate constant *k*_dep_) transfers polarizations from the radical cation to the α-amino alkyl radical in the same way, but also involves macroscopic chemical turnover. All three processes can be formulated as pseudo first-order ones because the concentration of **DH** is much higher than the concentrations of ”polarized molecules”.

Starting with neutral radicals (i.e., for the sensitizer XA), the observable must follow a simple first-order rate law. However, starting with radical cations (i.e., in the case of AQ), Scheme 2 predicts more complex kinetics comprising two exponential terms, with rate constants *k*_HT_ and (*k*_ET_ + *k*_dep_) and positive signs of both pre-exponential factors. Because the experimental results are evidently very well represented by a monoexponential decay (Figure 1), the data can only be accommodated by the intermediacy of radical-ion pairs in two limiting situations, where one of the exponential terms dominates. The first is that relayed deprotonation is slower than electron self-exchange, in which case *k*_ET_ becomes the observed rate constant; the second is that relayed deprotonation is slower than hydrogen self-exchange, in which case it limits the rate for the right-hand-side pathway from 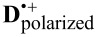 to **DH**_polarized_, and the observed rate constant is given by *k*_ET_ + *k*_dep_.

The absence of a residual polarization with AQ, which is clearly perceived in Figure 1, allows a decision between these two alternatives. When the free radicals 
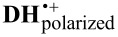
 do not undergo relayed deprotonation, all protons remain in place throughout the reaction sequence; furthermore, none of them are directly bound to centers bearing the unpaired electron, so relaxation losses will be extremely small. In this situation, one therefore expects complete cancellation of cage and escape polarizations, and no signal should remain on long time scales. Relayed deprotonation, however, removes one of the 12 polarized protons, which the subsequent hydrogen exchange replaces by an unpolarized one. Hence the escape polarization can only compensate 11*/*12 of the cage polarization, and a residual signal of a little more than 8 percent is expected. The fact that no such residual signal is observed militates for the unimportance of relayed deprotonation for the CIDNP kinetics.

With Equation 2, the free energy of the relayed deprotonation 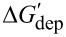 can be estimated from the reduction potential Φ_red_ (**DH**^•+^) of the radical cation (see, Table 1, but taken relative to NHE instead of SCE), the p*K*_a_ of the protonated amine 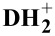 (8.9 [43]), and the calculated heats of formation ∆*H*_f_ of **D**^•^ (+208 kJ/mol) and **DH** (+87 kJ/mol):

[2]
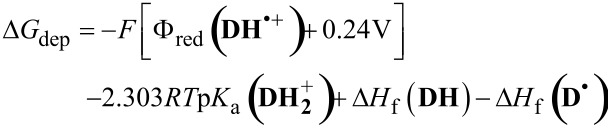


The obtained result, *−*8 kJ/mol, shows that, for this amine, relayed deprotonation is almost thermodynamically neutral. Newman projections further indicate relayed deprotonation to be sterically more demanding than hydrogen self-exchange, because two of the gauche interactions in the transition state are between larger groups in the former reaction compared to the latter. In combination with the lower rate in the XA-sensitized experiments, this lends further support to the presumption that *k*_dep_ is smaller than *k*_ET_.

The low value of 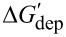 raises the possibility that, for DABCO, relayed deprotonation of **DH**^•+^ may be reversible. Starting with neutral radicals, the kinetic analysis would then be completely analogous to the above one, but with the roles of *k*_ET_ and *k*_HT_ interchanged. Because the preceding discussion has shown that relayed deprotonation should definitely be slower than electron self-exchange, the condition for a monoexponential decay is very likely to be fulfilled. Hence, the observed decay rate constant in the photoreaction with XA may well be a compound quantity, (*k*_HT_ + *k*_dep_). In that case, no residual CIDNP signal is expected because the α hydrogen of the amine is already removed in the cage, and the ketyl hydrogen, which re-enters the amine in the cage recombination, is easily exchangeable, so will not develop an appreciable polarization during the life of the radical pairs.

Nelsen et al. measured electron transfer rates for different redox couples and used the Marcus cross-rate theory to calculate self-exchange rate constants from these data; for DABCO, for which only one such couple was available, they reported a value of 7*.*3 *×* 10^3^ M*^−^*^1^s*^−^*^1^ [44]. This indirectly obtained electron-self-exchange rate constant is four orders of magnitude lower than even the smaller of our two directly observed rate constants, and under no circumstances could the curves of Figure 1 be reconciled with such a slow process. We have no explanation for that discrepancy but point out that it would be very surprising if an electron self-exchange (which is accompanied by comparatively small geometry changes and is less influenced by sterical constraints than bond-forming reactions, because it can occur over longer distances than the contact distance [45]) should be so strongly decelerated in relation to the other chemical processes taking place in that sterically hindered system, namely, hydrogen self-exchange and almost energetically neutral proton transfer.

**Figure 3:**
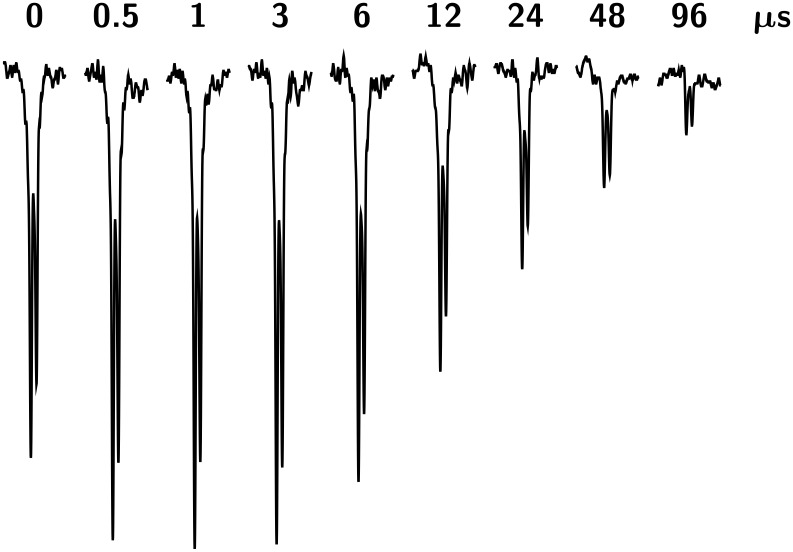
Time-resolved CIDNP signals of the 18 equivalent β protons (d, 0.98 ppm) of triisopropylamine (TIPA) in the photoreaction with xanthone (XA) as functions of the delay (given at the respective trace) between the laser flash and the sampling NMR pulse. Note the exponential increase of the delay with the spectrum number. Excitation intensity, ca. 80 mJ per flash; NMR pulse width, 1*.*0 µs; temperature, 279 K; amine concentration, 5*.*0 *×* 10*^−^*^4^ M; sensitizer concentration, 1 *×* 10*^−^*^3^ M. Further explanation, see text.

Figure 3 displays the outcome of a time-dependent CIDNP experiment on TIPA sensitized by XA. This amine exhibits the peculiarity that its CIDNP spectra are completely dominated by the emissive doublet of the β protons at 0.98 ppm; in the example, the α protons (septet at 3.13 ppm) bear no discernible polarization whatsoever, and with the sensitizer AQ, their CIDNP signals are so tiny as to prohibit any interpretation. In contrast to DABCO, the CIDNP phases with TIPA are emissive regardless of the sensitizer, and there is a much more noticeable initial increase of the magnitude of the polarizations.

However, the striking influence of the sensitizer is observed also for this amine, and is even stronger than for DABCO: With AQ as compared to XA, the polarizations of TIPA decrease faster by a factor of five at room temperature. The intermediacy of different radical pairs, and thus of different initial free radicals again provides a natural explanation.

The β protons must have a positive hyperfine coupling constant both in the radical cation and in the α-amino alkyl radical of TIPA. The observed emissive polarization thus constrains the amine-derived radical to have a lower *g* value than the sensitizer-derived radical. For the sensitizer XA this in only possible in the case of the neutral radical pairs because the *g* value of the amine radical cation (2.0037 [46]) is higher than the *g* values of the sensitizer radical anion and ketyl radical (2.0036 and 2.00345 [5]), while for the α-amino alkyl radical of a saturated monoamine a *g* value of 2.0030 is typically expected [16]. The polarization phases of this substrate thus further support the intermediacy of a radical ion pair with AQ and of a pair of neutral radicals with XA.

For a pair of neutral radicals, the absence of CIDNP of the α protons can be understood because in the radical pair this proton is attached to the oxygen of the sensitizer ketyl radical and is, therefore, exchangeable, so cannot pick up a noticeable polarization during the pair life. For a radical ion pair, the α protons should exhibit only small polarizations, because the ratio of the hyperfine coupling constants of the α and β protons in the radical cation of TIPA is much smaller (about 2:1 [46]) than in a less strained amine, such as triethylamine, the number of α protons is six times smaller, and the signal splitting into a septet causes the individual lines to be concomitantly lower in intensity.

Because the observed β protons remain in place during all transformations from the radical pairs over the free radicals to the diamagnetic product **DH**, a residual polarization on long time scales is neither expected nor found. The initial signal growth in Figure 3 is due to radical-pair formation, i.e., to the quenching of the sensitizer triplet by the amine. We emphasize that the resulting biexponential rate law is characterized by opposite signs of the two pre-exponential factors as opposed to the equal signs that result from the decay kinetics of Scheme 2. In principle, the quenching rates could be extracted from the signal rise, but that determination is not very accurate under conditions best suited for investigating the self-exchange [34]. The decay of the signal is once more excellently described by a single exponential, with the same implications for the mechanism as in the case of DABCO.

The free energy for the deprotonation of the radical cation by the amine itself can again be calculated with Equation 2. The p*K*_a_ value of 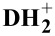 is only known in aqueous diglyme, where it is between 6.9 and 9.2, depending on the water content [47]. Even with the smaller of these values, using the reduction potential of Table 1 and the calculated heats of formation (∆*H*_f_ (**D**^•^), *−*33 kJ/mol; ∆*H*_f_ (**DH**), *−*111 kJ/mol) we arrive at an exergonicity of at least *−*50 kJ/mol, which means that a reversibility of that deprotonation can be discounted. Sterically, that deprotonation is much more demanding than the hydrogen self-exchange, as Newman projections of the expected transition states show. With both reactions, there are four gauche interactions between methyl and a large group (iPr or N(iPr)_2_), whereas the other two gauche interactions are between the two large groups iPr and N(iPr)_2_ in the former reaction, but only between two methyl groups in the latter. On these grounds, we assume that, notwithstanding the substantial driving force, *k*_dep_ is smaller than *k*_PT_ for this amine. That assumption immediately leads to the consequence that the rate constant in the reaction with AQ, i.e., the compound quantity (*k*_ET_ + *k*_dep_), should again be dominated by *k*_ET_ because the rate constant *k*_PT_, which is measured in the XA-sensitized reaction, is five times smaller than the observed rate constant in the AQ-sensitized reaction.

**Figure 4:**
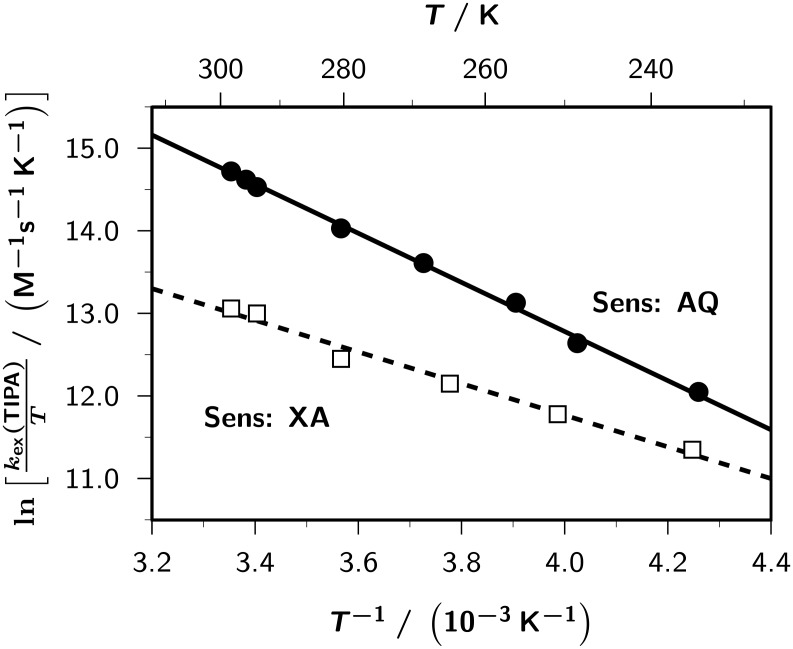
Eyring plots for the self-exchange rate constants *k*_ex_ (TIPA) of triisopropylamine (TIPA) sensitized by anthraquinone (AQ) (full circles and solid line; linear regression, 24*.*68 *−* 2975/*T* ) and xanthone (XA) (open circles and broken line; linear regression, 19*.*43 *−* 1917/*T* ). All experimental parameters, expect for the temperature *T*, as in Figure 3. Further explanation, see text.

Experimental activation parameters should thus be meaningful for the presumed single reaction in each of these cases (sensitizer AQ, pure electron self-exchange; sensitizer XA, pure hydrogen self-exchange). Eyring plots are displayed in Figure 4. Their good linearity also in the AQ-sensitized reaction lends a posteriori support to our above reasoning.

From the regression lines in Figure 4, one calculates the activation parameters given in Table 2. The much more negative activation entropy in the XA-sensitized reaction is clearly consistent with our preceding explanation of the different reaction rates, because a hydrogen self-exchange must involve a more ordered transition state than an electron self-exchange; the small positive activation entropy in the AQ-sensitized reaction also supports our interpretation, because this is a well-known fact for outer-sphere electron transfer reactions of organic compounds in polar solvents [48].

**Table 2:** Activation parameters for the self-exchange reactions of TIPA, as obtained from the Eyring plots of Figure 4.

Reaction	∆*H*^‡^ (kJ/mol)	∆*S*^‡^ (J K*^−^*^1^ mol*^−^*^1^)	∆*G*^‡^_298_ (kJ/mol)

**DH**^•+^ + **DH**	24.7	+7*.*6	22.4
**D**^•^ + **DH**	15.9	*−*36*.*0	26.6

According to the Marcus theory [49], the free energy of activation of an electron-self-exchange reaction equals one quarter of the reorganization energy λ, which is the energy needed to distort the geometries of the reactants (inner reorganization energy λ_i_) and of the surrounding solvent shell (outer reorganization energy λ_o_) to the geometries of the products including their solvent shell, but without transferring the electron. For organic compounds, λ_o_ typically dominates, with λ_i_ rarely accounting for more than 15% of the total reorganization energy [50]. For an estimate of λ, we therefore neglect λ_i_.

Using a continuum model, Marcus [49] has derived the following expression for λ_o_ for a self-exchange of a singly charged ion with its neutral parent compound,

[3]
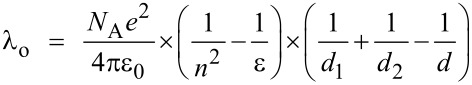


where *N*_A_, *e*, and ε_0_ are Avogadro’s number, the electron charge, and the vacuum permittivity, *n* and ε are the index of refraction and the relative permittivity of the solvent, *d*_1_ and *d*_2_ are the molecular diameters of the two reactants, and *d* is their encounter distance. For acetonitrile, the polarity parameter (1*/n*^2^ *−* 1*/*ε) amounts to 0.527 at room temperature. The calculated shapes of **DH** and **DH**^•+^ are practically identical for our amines, and the geometries of both TIPA and DABCO deviate only slightly from spherical; they are are oblate spheroids with the shorter semi-axis orientated perpendicular to the plane of the three methine protons in the former case and along the N–N axis in the latter. To get the molecular dimensions, we added the van-der-Waals radii of the respective outermost atoms along each semi-axis (N, 1.55 Å [51] for the shorter semi-axis of DABCO; H, 1.1 Å [52] for all other semi-axes) and averaged the three diameters. Identifying the shorter diameter with the encounter distance, we arrive at an outer-sphere reorganization energy of 82*.*6 kJ/mol for TIPA (*d*_1_ = *d*_2_ = 7*.*5 Å; *d* = 6*.*5 Å), i.e., a value of 20*.*6 kJ/mol for 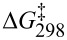; if the encounter distance, which is not precisely known, were identical to the average molecular diameter 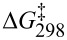 would increase to 24.4 kJ/mol. In view of this uncertainty, an elaborate computation of λ_i_ is not justified, but the calculated activation parameter is seen to be in very good agreement with the experimental result of Table 2. Finally, the slightly smaller molecular size of DABCO (*d*_1_ = *d*_2_ = 6*.*2 Å; *d* = 5*.*7 Å) leads to a computed increase of 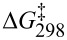, relative to TIPA, by 6.3 kJ/mol, corresponding to a decrease of the self-exchange rate constant by a factor of 12.7, which at first glance does not seem to compare very favourably with the experimental ratio (5.3) observed in this work; however, DABCO has two nitrogen sites that can participate in the exchange, and taking this into account by multiplying the experimental ratio by 2 reduces the discrepancy to less than 20 percent.

## Conclusion

The described direct measurements of self-exchange rate constants by time-resolved photo-CIDNP experiments have made it possible to distinguish between the different types of free radicals occurring in these systems, and have corroborated and complemented our previous [5–7] mechanistic findings on sensitized photoreactions of tertiary aliphatic amines. It would have been difficult or impossible to obtain these results by other techniques because no other kind of spectroscopy attaches labels (the polarizations) at the stage of the intermediates and observes them in the products, thus highlighting the interconnections of species along the reaction coordinate. The present study thus again demonstrates the power of CIDNP to provide detailed insights into complex reaction mechanisms.

## Experimental

TIPA was synthesized and purified according to a literature procedure [47]; DABCO and all the sensitizers were commercially obtained and purified by double sublimation. The purchased solvent acetonitrile-*d*_3_ was carefully dried to a water content of less than 5 *×* 10*^−^*^4^ M in a specially designed apparatus [53]. Sensitizer concentrations were chosen to give an extinction of about 1 in a 5 mm NMR tube. ^1^H CIDNP experiments were carried out on a Bruker WM 250 NMR spectrometer with a special probe [34] allowing side-on illumination of the samples. The temperature in the probe was controlled to *±*0*.*3 K. Presaturation sequences [31] were used to remove unchanging background magnetization. The light source was a Lambda Physik EMG 101 laser (XeCl, 308 nm, 15 ns pulse width, *±*5 ns jitter, *±*3% energy fluctuations), which was triggered by the acquisition system of the spectrometer.

## References

[1] Schaefer C G, Peters K S (1980). J Am Chem Soc.

[2] Simon J D, Peters K S (1981). J Am Chem Soc.

[3] Inbar S, Linschitz H, Cohen S G (1981). J Am Chem Soc.

[4] Miyasaka H, Morita K, Kamada K, Mataga N (1990). Bull Chem Soc Jpn.

[5] Goez M, Sartorius I (1993). J Am Chem Soc.

[6] Goez M, Sartorius I (1994). Chem Ber.

[7] Goez M, Sartorius I (2003). J Phys Chem A.

[8] Pischel U, Zhang X, Hellrung B, Haselbach E, Muller P-A, Nau W M (2000). J Am Chem Soc.

[9] Pischel U, Nau W M (2001). J Am Chem Soc.

[10] Steiner U E, Ulrich T (1989). Chem Rev.

[11] Goez M, Neckers D C, Volman D H, von Bünau G (1997). Photochemically Induced Dynamic Nuclear Polarization. Advances in Photochemistry.

[12] Goez M (2009). Annu Rep NMR Spectrosc.

[13] Goez M, Forbes M D (2010). Carbon-Centered Free Radicals and Radical Cations: Structure, Reactivity, and Dynamics.

[14] Berliner L J, Bagryanskaya E, Misra S K (2011). Multifrequency Electron Paramagnetic Resonance.

[15] Goez M (2012). Top Curr Chem.

[16] Roth H D, Manion M L (1975). J Am Chem Soc.

[17] Schäublin S, Wokaun A, Ernst R R (1977). J Magn Reson.

[18] Closs G L, Miller R J (1979). J Am Chem Soc.

[19] Goez M, Kuprov I, Hore P J (2005). J Magn Reson.

[20] Goez M, Kuprov I, Mok K H, Hore P J (2006). Mol Phys.

[21] Griller D, Howard J A, Marriott P R, Scaiano J C (1981). J Am Chem Soc.

[22] Bock H, Göbel I, Havlas Z, Liedle S, Oberhammer H (1991). Angew Chem, Int Ed Engl.

[23] Fickling M M, Fischer A, Mann B R, Packer J, Vaughan J (1959). J Am Chem Soc.

[24] Given P H, Peover M E, Schoen J (1958). J Chem Soc.

[25] Hub W, Schneider S, Dörr F, Oxman J D, Lewis F D (1984). J Am Chem Soc.

[26] (2009). Gaussian 09.

[27] Herkstroeter W G, Lamola A A, Hammond G S (1964). J Am Chem Soc.

[28] Scaiano J C (1980). J Am Chem Soc.

[29] Saltiel J, Curtis H C, Metts L, Miley J W, Winterle J, Wrighton M (1970). J Am Chem Soc.

[30] Closs G L, Sitzmann E V (1981). J Am Chem Soc.

[31] Goez M, Mok K H, Hore P J (2005). J Magn Reson.

[32] von Raumer M, Suppan P, Haselbach E (1996). Chem Phys Lett.

[33] Goez M (1990). Chem Phys Lett.

[34] Goez M (1990). Chem Phys.

[35] Goez M, Eckert G (1993). Z Phys Chem (Muenchen, Ger).

[36] Kaptein R (1971). J Chem Soc, Chem Commun.

[37] Muto Y, Nakato Y, Tsubomura H (1971). Chem Phys Lett.

[38] Kaise M, Someno K (1987). Chem Lett.

[39] Aarons L J, Adam F C (1972). Can J Chem.

[40] Sieiro C, Sanchez A, Crouigneau P (1984). Spectrochim Acta, Part A.

[41] Sharpless N E, Bradley R B, Ferretti J A (1974). Org Magn Reson.

[42] Wilson R (1968). J Chem Soc B.

[43] Castro E A, Aliaga M, Campodonico P R, Leis J R, García-Río L, Santos J G (2008). J Phys Org Chem.

[44] Nelsen S F, Weaver M N, Luo Y, Pladziewicz J R, Ausman L K, Jentzsch T L, O’Konek J J (2006). J Phys Chem A.

[45] Kavarnos G J, Turro N J (1986). Chem Rev.

[46] de Meijere A, Chaplinski V, Gerson F, Merstetter P, Haselbach E (1999). J Org Chem.

[47] Kuffner F, Koechlin W (1962). Monatsh Chem.

[48] Ghorai P K, Matyushov D V (2006). J Phys Chem A.

[49] Marcus R A (1964). Annu Rev Phys Chem.

[50] Jensen B S, Ronlán A, Parker V D (1975). Acta Chem Scand, Ser B.

[51] Bondi A (1964). J Phys Chem.

[52] Rowland R S, Taylor R (1996). J Phys Chem.

[53] Goez M (1998). J Magn Reson.

